# Exercise and Garlic Modulate microRNAs Involved in Diabetic Cardiopathy

**DOI:** 10.5935/abc.20180259

**Published:** 2019-02

**Authors:** Aline Regina Ruiz Lima

**Affiliations:** Faculdade de Medicina de Botucatu - Universidade Estadual Paulista (UNESP) - Botucatu, SP - Brazil

**Keywords:** Diabetes Mellitus/complications, Exercise/prevention & control, MicroRNAs, Heart Diseases, Angiogenesis Inducing Agents

Diabetes mellitus (DM) is a major risk factor for cardiovascular disorders and stroke development and is associated with increased morbidity and mortality.^1^ The prevalence of diabetes is increasing at an alarming rate worldwide. Indeed, according to estimates of the International Diabetes Federation, 552 million people are expected to be diabetic in 2030.^2^ Although a definitive cure is not on the horizon, with proper management, diabetic patients can attenuate the development of serious complications that reduce life quality and expectancy. Facing a considerable rate of occurrence and prognosis complications, studies focusing on high efficiency and low toxicity treatments are of great importance.^3^

MicroRNAs are small non-coding RNAs controlling gene expression and participating in many physiopathological processes. These small molecules are getting a lot of attention nowadays since they are universally recognized as major regulators of gene expression and as key controllers of several biological and pathological processes.^4^ They are essential intracellular mediators in a variety of cellular processes, such as inflammation, mitochondrial metabolism, apoptosis, among others. Therefore, miRNAs could be potential targets to treat some chronic diseases. Besides, these molecules can also be used as early biomarkers, once they are released in urine and blood when in presence of tissue lesion.^5^ Recently, it was verified that miRNAs are also involved in cardiovascular disorders, especially those which impaired angiogenesis is observed.^6^

Considering this scenario, Mostafa et al.^7^ evaluated the effects of garlic consumption and voluntary exercise, alone and together, on microRNAs 126 and 210, involved in cardiac angiogenesis, in diabetic rats.

Garlic, *Allium sativum L*, is commonly used in traditional phytotherapy and there are many studies showing its beneficial effects in several disorders, such as cancer, cardiovascular diseases and diabetes. Also, some authors already showed its effects in angiogenesis.^8^ Indeed, Mostafa et al.,^7^ found that diabetes reduced cardiac angiogenesis and garlic consumption increased this angiogenesis in diabetic rats.

Aerobic exercise is a non-pharmacological therapeutic approachable to improve cardiovascular health in general. Regular practice of exercises results in several health benefits, such as improvement in body composition, physical capacity, insulin resistance, endothelial function, arterial hypertension, and quality of life.^9^ Besides these benefits, exhaustive exercise practice can contribute to oxidative stress, producing reactive oxygen species (ROS). In animal models, some authors believe that voluntary exercises could show more positive effects.^10^ In fact, Mostafa et al.^7^ observed that voluntary exercises reduced triglycerides and LDL cholesterol serum levels and enhanced HDL serum levels and HDL/LDL ratio in comparison to the diabetic control group.

In Mostafa study,^7^ miRNAs 126 expression is reduced in diabetic rats. Both treatments, physical exercise or garlic ingestion, were able to increase its expression. Interestingly, when taken together, exercise and garlic, there was an additional increase in miRNA 126 expression. MicroRNA 126 is endothelium-specific, modulating angiogenesis and contributing to endothelium homeostasis. Possibly, miRNA 126 acts through inhibition of negative regulators of VEGF pathway.^11^

In response to hypoxia conditions, endothelium cells increase miRNA 210 expression to promote angiogenesis. In the same way, other authors have described the high expression of this miRNA in hyperglycemia contexts, such as diabetes.^12^ These studies corroborate Mostafa et al. results, that showed increased miRNA 210 in diabetic rats. This expression was reduced with both treatments, voluntary exercise or garlic consumption, and there was a bigger reduction when taken together.

It is well known that physical exercise has positive effects in controlling glycemia levels. Moreover, practice of physical exercise is recommended to good health maintenance and quality of life.^13^

A systematic review of garlic effects on lipidic and glucose parameters in diabetic patients was recently published. The authors concluded that garlic can reduce lipid profile as well as glucose parameters and be therapeutically effective in patients with cardiovascular diseases and diabetes.^14,15^

Some of those positive effects obtained by physical exercise and garlic ingestion may be to modulation of specific microRNAs, according to Mostafa and collaborators. It is interesting to observe that the response to those treatments was amplified when they were combined, almost like an adjuvant effect.

Although these promising and interesting results, more studies on what mechanisms and which intracellular pathways modulate microRNAs expression involved in the cardiac angiogenesis and lipidic profile improvement provided by voluntary physical exercise and garlic consumption in diabetes mellitus are necessary.
